# Pathogenesis and vertical transmission of a transplacental rat cytomegalovirus

**DOI:** 10.1186/1743-422X-3-42

**Published:** 2006-06-01

**Authors:** Hwei-San Loh, Mohd-Azmi Mohd-Lila, Sheikh-Omar Abdul-Rahman, Lik-Jun Kiew

**Affiliations:** 1 Institute of Bioscience, Universiti Putra Malaysia, Serdang, Selangor, Malaysia; 2 Department of Pathology and Microbiology, Faculty of Veterinary Medicine, Universiti Putra Malaysia, Serdang, Selangor, Malaysia

## Abstract

**Background:**

Cytomegalovirus (CMV) congenital infection is the major viral cause of well-documented birth defects in human. Because CMV is species-specific, the main obstacle to developing animal models for congenital infection is the difference in placental architecture, which preludes virus transmission across the placenta. The rat placenta, resembling histologically to that of human, could therefore facilitate the study of CMV congenital infection in human.

**Results:**

In this report, we present clear evidences of the transplacental property of a new rat CMV (RCMV), namely ALL-03, which had been isolated from placenta and uterus of the house rat. Our study signifies the detection of infectious virus, virus particles, viral protein and DNA as well as immune response to demonstrate a natural model of acute CMV infection including the immunocompetent and immunocompromised host associated with or without pregnancy. It is characterized by a full range of CMV related clinical signs; lesions and anatomical virus distribution to uterus, placenta, embryo, fetus, neonate, lung, kidney, spleen, liver and salivary gland of the infected rats in addition to the virus-specific seroconversion. The preference of the virus for different organs mimics the situation in immunocompromised man. Most interestingly, the placenta was observed to be involved in the maternofetal infection and hence confirmed the hypothesis that the RCMV strain ALL-03 is capable to cross the placenta and infect the offsprings congenitally.

**Conclusion:**

The maternal viremia leading to uterine infection which subsequently infecting to the fetus through the placenta is the most likely phenomenon of CMV vertical transmission in our study.

## Background

Cytomegalovirus (CMV) infection is the most frequent congenital infection in humans worldwide, with an incidence of 0.2–2.2% of live births [1,2]. One major concern of CMV congenital infection is birth defects including mental retardation, microcephaly, epilepsy, and blindness. However, little is known on how the virus is transmitted to the fetus during pregnancy [3]. The possible routes of transmission of human CMV (HCMV) to the offsprings are vertical via germ line cells or transplacentally; perinatally and postnatally. There are several reports strongly supporting the hypothesis that placental infection precedes viral transmission to the fetus [3-6].

Due to the strict species-specificity of HCMV, it has not generally been possible to study this virus in experimental animals. A number of natural CMV infections in various animal species have been utilized for modeling HCMV infection. Among the animal CMVs, transplacental transmission has been reported for rhesus macaque CMV [7], porcine CMV [8] and guinea pig CMV (GPCMV) [9]. However, the expenses of the primates and pigs, as well as the rarity of their CMV seronegative animals make these models impractical for large-scale vaccine studies. For these reasons, rats, mice, and guinea pigs came into favor because of their small size, low cost, short life span, ease of handling and high reproductive rate. More importantly, these CMVs (RCMV, MCMV and GPCMV) closely resemble HCMV. For studying the transplacental hypothesis, it is important to consider the great diversity in the placental structures among human and model. Favorably, these three animals have similar discoidal hemochorial placentation to that of human [10]. However, none of the existing MCMVs and RCMVs demonstrated a clear involvement of the placenta in vertical transmission [11,12] and are therefore, less suitable for the study of CMV congenital infection [13,14]. Although GPCMV provides a well-characterized model of transplacental viral infection, studies in this system have been hampered by a lack of genetic knowledge of the animal itself. In addition, the cost of guinea pigs is less practical for large-scale vaccine and long-term maintenance studies as compared to mice and rats. Meanwhile, the desirable features of rat biology include more human-like physiological responses for disease process, an extensive behavioral database, and larger size (better suited to surgical manipulation and repeated blood sampling) are the major advantages of the rat model over the mouse model. Besides, following human [15,16] and mouse [17], rat is the third mammalian for which the complete genome has been determined. Almost all human genes noted to be associated with disease have known counterparts in the rat genome [18]. This genetic explorer for the rat provides an unprecedented opportunity to take advantage of the rich and robust history of experimental studies utilizing this species to study HCMV disease. Hence, the rat system is a significant advance on the guinea pig or mouse model for studying various aspects of viral pathogenesis, the effect of therapeutic intervention as well as the evaluation of vaccine candidates for CMV congenital infection in humans.

In our previous study, we have discovered a new RCMV isolate (ALL-03) obtained from placenta and uterus of the house rat, *Rattus rattus diardii *[19]. The involvement of the placental and uterine tissues during virus isolation indicates that the virus has the ability to cross the placenta and infect the fetus. Therefore, an attempt was made to study the maternofetal involvement in the pathogenicity of RCMV infection. In this report, we demonstrate a natural model of acute RCMV infection, which includes the characteristic organ distribution of RCMV in male rats and female rats with or without pregnancy as well as the immune response to the infection. More importantly, this is the first RCMV infection study capable of presenting a clear evidence of transplacental transmission in pregnant rats.

## Results

The rats were challenged with RCMV and sampled at different time point, i.e. day 21 p.i. for Experiment A, B and D, meanwhile, day 13–14 p.i. for Experiment C. The presences of infectious virus, viral DNA and antigen, virus particles as well as seroconversion were assessed by employing techniques such as histological and immunohistological stainings including H&E, IIP and IIF; virus assay; protein blotting; PCR; TEM and indirect ELISA.

### Clinical observation

The animals in the four experiments were observed twice daily until the time for sampling. No abnormality was observed in all control groups throughout the study. All treatment groups showed no clinical signs from day 1 to day 5 p.i. After an incubation period of 6 to 21 days, the RCMV infection became symptomatic especially the immunocompromised groups. The infected rats of all immunocompromised groups in Experiment A, B, C and D as well as immunocompetent groups in Experiment C and D became less active. The clinical signs such as hemorrhages at the extremities of the limbs and tails, and ruffling of hair coat were obvious. There were absences of abortion and mortality in rats up to day 21 p.i. The post-partum neonates in Experiment C did not show any apparent abnormality as compared to the control groups except the litter size in treatment groups (7–8 pups) was slightly smaller than that of control groups (8–9 pups).

### Gross pathology

No abnormalities were observed in the organs of all control animals in the four experiments. The lesions such as congestion of renal cortex and corticomedullary junction, generalized hemorrhage of the lung and marked splenomegaly were common and observed mostly in immunosuppressed and pregnant rats. Mild hemorrhage was found in the uterus serosal surface of an infected immunosuppressed dam (Experiment D) carrying seven conceptuses.

### Histological and immunohistological pathology

The presences of the characteristic histopathological changes in the organs of animals in the four experiments were determined by H&E staining and further confirmed by IIP test. No specific lesions caused by RCMV disease were observed in all control groups. The organs that appeared normal histologically and did not show characteristics of infection in all treatment groups were brain, heart, testes and ovary. The immunoreactivity of IIP test of the treatment groups is presented in Table 1. The histopathological and immunopathological findings are described in the following:

**Table 1:** Positive immunoreactivity of IIP test on different tissue sections of treatment groups.

**Experiment/Organ**	**A (Day 21 p.i.)**		**B (Day 21 p.i.)**		**C (Day 13–14 p.i.)**		**D (Day 21 p.i.)**	
	
	**Group**							
	
	**v**	**pv**	**v**	**pv**	**v**	**pv**	**v**	**pv**
Brain	0/3	0/3	0/3	0/3	0/3	0/3	0/3	0/3
Salivary gland	0/3	1/3	0/3	1/3	0/3	0/3	1/3	2/3
Heart	0/3	0/3	0/3	0/3	0/3	0/3	0/3	0/3
Lung	0/3	2/3	1/3	3/3	2/3	3/3	3/3	3/3
Spleen	0/3	1/3	0/3	2/3	2/3	3/3	2/3	3/3
Liver	0/3	1/3	0/3	2/3	1/3	3/3	1/3	3/3
Kidney	0/3	2/3	0/3	2/3	2/3	3/3	2/3	3/3
Testes	0/3	0/3	-	-	-	-	-	-
Ovary	-	-	0/3	0/3	0/3	0/3	0/3	0/3
Uterus	-	-	1/3	3/3	2/3	3/3	3/3	3/3
Neonate	-	-	-	-	6/15	12/15	-	-
Placenta	-	-	-	-	-	-	12/15	8/10
Fetus	-	-	-	-	-	-	9/15	6/10
Embryo*	-	-	-	-	-	-	-	5/5

Note: Abbreviations: v = virus-infected and pv = virus-infected with immunosuppression.

* = eroded placenta and developing embryo in uterus at ≤ 7 days of pregnancy.

0/3 = no detectable positive result over triplicate sample trials in all three rats.

#### Salivary gland

**Figure 1:**
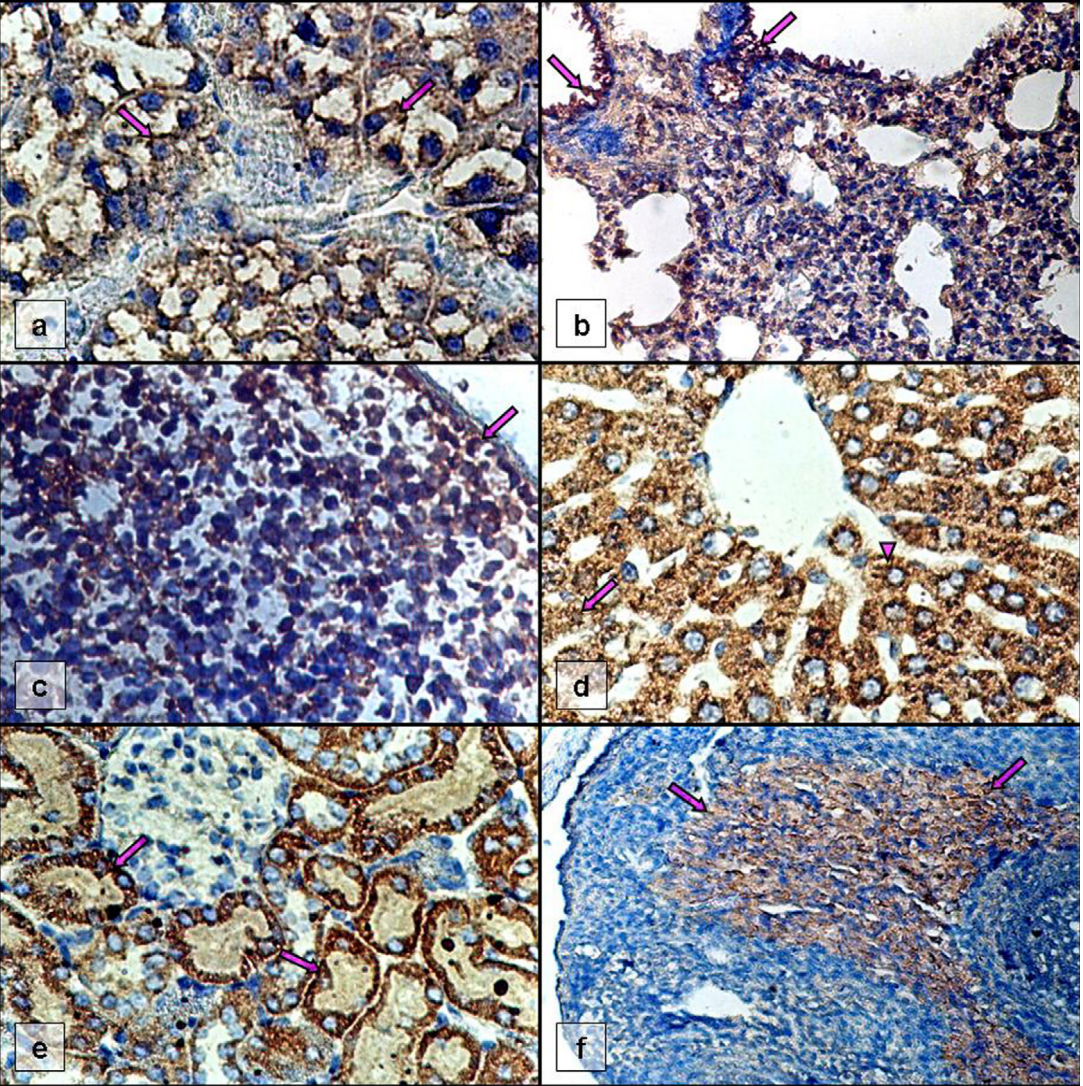
**Positive IIP-stained tissue sections of infected immunosuppressed rats**. (a) secretory acinar cells (arrows) of sublingual gland (D; day 21 p.i.; × 400), (b) bronchioles (arrows) and lung parenchyma (D; day 21 p.i.; × 200), (c) splenic cells (arrow; D; day 21 p.i.; × 400), (d) nucleus (arrow) and cytoplasm (arrowhead) of hepatocytes (C; day 13 p.i.; × 400), (e) renal tubules (arrows; D; day 21 p.i.; × 400), (f) stratum basalis (arrows) of endometrium (C; day 13 p.i.; × 200).

Localization of RCMV infection in all salivary glands, i.e. parotid, submandibular and sublingual glands was observed. However, the submandibular gland was stained more frequently than the other types of salivary glands. The positive findings were established in immunosuppressed rats in Experiment A and B; in pregnant rats of both treatment groups in Experiment D. No positive features of RCMV infection in all groups of Experiment C were evident. The RCMV infection in the salivary glands was confined to the striated ducts, secretory acini (Figure 1a) and trabeculae connective tissues. The histological abnormalities such as the swollen and enlarged mucous cells and acinar cells were evident but not frequently.

#### Lung

The parenchyma particularly the bronchioles and alveoli was solely permissive for CMV infection (Figure 1b). Intranuclear and intracytoplasmic inclusion bodies stained extensively by IIP were found in the swollen bronchiolar and alveolar cells. The macrophages and occasional pneumocytes in alveolar wall as well as ciliated bronchiolar epithelia were immunoreactive to CMV. The common pathological features included the congested and hemorrhagic interstitium, accumulation of proteinaceous fluid with infected and uninfected monocytes and macrophages in alveoli and bronchioles, thickened alveolar septa, perivascular inflammatory cell cuffings and lymphocytic hyperplasia.

#### Spleen

Some of the infected immunocompetent animals showed reactive hyperplasia of spleens though IIP test did not show positive staining. In contrast, the splenic tissue of immunosuppressed animals especially those with splenomegaly was notably stained by IIP (Figure 1c). Most of the infected areas were less extensive and often scattered at a distance in red pulps. The periarterior lymphocyte sheaths of immunosuppressed animals had shrunk to some extent. The splenic sinusoids were infiltrated with numerous macrophages, many of which contained viral antigens. Numerous lymphocytes and plasma cells were often present in both white and red pulps.

#### Liver

The intensity of immunostaining was marked in liver tissues of immunosuppressed animals in Experiment C, which involved almost entirely the tested sections (two cases; Figure 1d). Most of the immunoreactive cells were located in the liver lobules adjacent to the capsule. Numerous hepatocytes showed characteristic inclusion bodies. The hepatocytes and many Kupffer cells contained viral antigens. The cytoplasm of hepatocytes stained more frequently than the nucleus (Figure 1d). The parenchyma showed patchy necrosis and degeneration. Hepatitis seen as infiltration of inflammatory cells in the parenchyma was one of the lesions found.

#### Kidney

Almost all treatment groups had animal(s) with signs of infection except the immunocompetent groups in Experiment A and B. In the kidney, infected cells were seen in both the cortex and medulla regions whereby the cortex region adjacent to the renal capsule was predominantly infected. Viral antigens were profound in the proximal and distal tubules, loop of Henle, and collecting tubules (Figure 1e), but less intensive in the renal corpuscles. The infection was predominant in cytoplasm rather than the nucleus. The mesangial cells were swollen and displayed characteristic nuclear inclusions, which contained the viral antigens. Tubulonephrosis in the form of ballooning degeneration was evident. Hypercellularity of the glomerulus was one of the lesions showing adhesion between the glomerular tuft and Bowman's capsule.

#### Uterus

All immunosuppressed female rats in the three experiments (B, C and D) regardless of presence or absence of pregnancy demonstrated signs of infection in particularly the endometrium. The immunoreactive cells were found majority in the stroma and surface epithelia, i.e. stratum basalis and stratum functionalis. The predominant localization of viral antigen was slightly different from one rat to another even within a group receiving identical treatment. Two rats in Experiment B and one in Experiment C had positive stromal cells for the immunostaining but not epithelial cells of glands. Meanwhile, three pregnant rats in Experiment D had viral tropism in epithelial cells only. Nevertheless, the majority of the rats showed immunoreactivity in the two regions and with more extensive staining in the stratum functionalis and stratum basalis (Figure 1f).

#### Placenta

**Figure 2:**
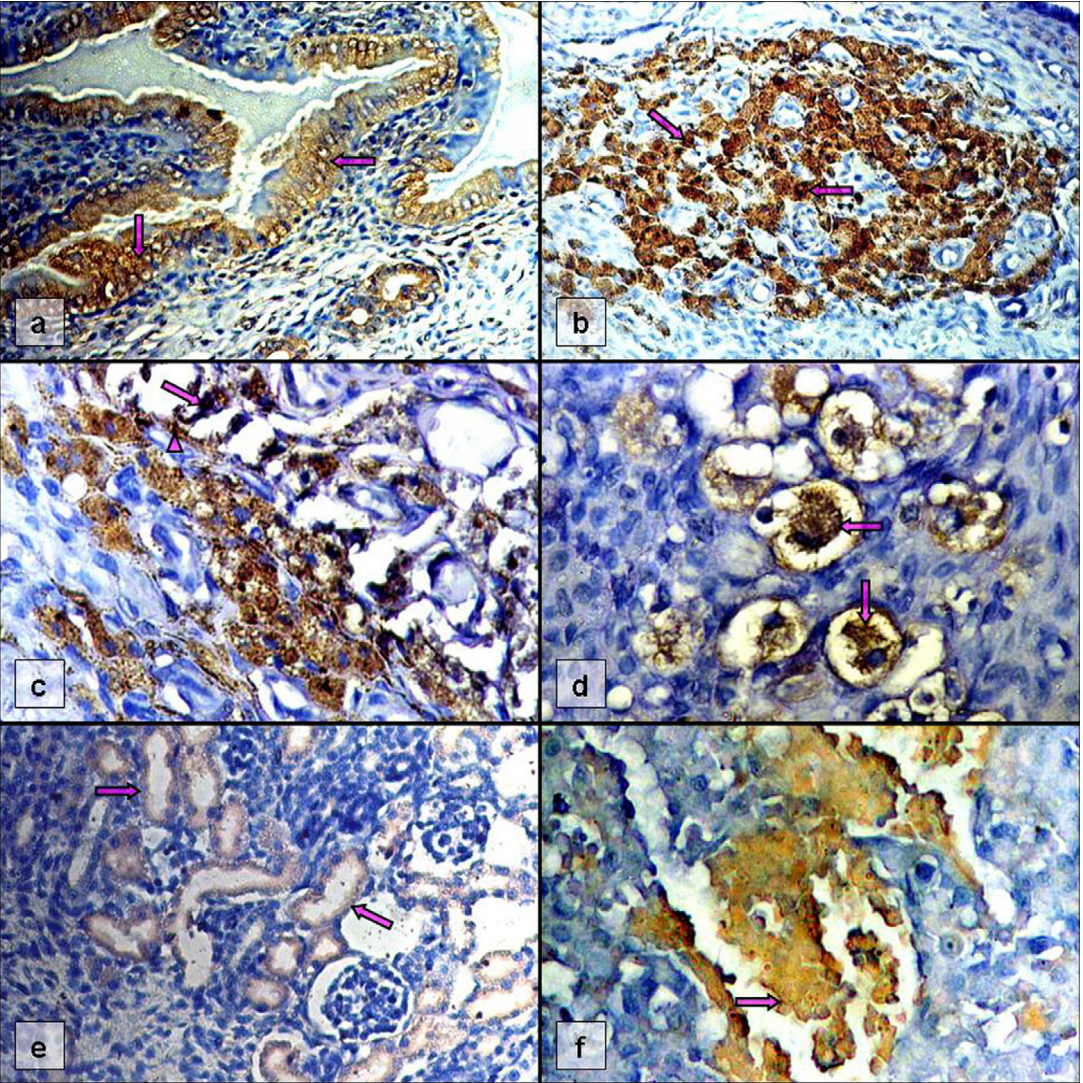
**Positive IIP-stained placental and fetal tissue sections of infected immunosuppressed dams**. Seven-day old placenta (D; day 21 p.i.): (a) decidual epithelia (arrows; × 200), (b) junctional zone (arrows; × 200), (c) chorionic villi (arrow) anchored to the decidual basalis concomitantly passing infection to junctional zone (arrowhead; × 400), and (d) trophoblast cells (arrows) in labyrinth zone (× 400); (e) fetal renal tubules (arrows) of 17-day pregnancy (D; day 21 p.i.; × 200), (f) fetal liver (arrow) of 18-day pregnancy (D; day 21 p.i.; × 400).

Both immunocompetent and immunosuppressed groups in Experiment D gave 80% of positivity in IIP staining. Meanwhile, the placenta sections (categorized as Embryo* in Table 1) of the two dams with about 7-day pregnancy, gave the most intensive stains i.e. 100% of positivity, which far surpassed those with pregnancy length greater than 14 days. The immunoreactive sites of the placenta were mostly at the decidual basalis, junctional zone and labyrinth zone (Figure 2a, 2b, 2c, 2d) but scarcely in the embryonic sites. However, the placenta with shorter gestation period showed more signs of infection in decidual basalis and junctional zone as compared to those with longer gestation period by which infections were found in the labyrinth zone predominantly. The chorionic villi anchoring to the decidual basalis concomitantly passing infection to junctional zone of placenta was observed (Figure 2c). These cells of maternal (decidual basalis) and fetal (chorionic villi and junctional zone) portions of placenta, were confirmed to be infected. The infected regions were found to be associated with intranuclear and intracytoplasmic inclusion bodies mostly of trophoblast cells in junctional and labyrinth zones (Figure 2b, 2d).

#### Neonate and fetus

The fetal tissues of those dams beyond 14 days of pregnancy in Experiment D, especially liver and kidney showed a significant presence of viral antigen (Figure 2e, 2f). For neonatal rats, no immunoreactivity was observed in salivary gland, however, positive results were found in the kidney and liver. The renal tubules were stained more frequently than the glomeruli. The proportion of immunoreactivity in a tissue was found generally greater in fetus rather than neonate.

### Virus assay

Virus was isolated from tissues of animals in Experiment C and D, namely the uterus, placenta, embryo, neonate and fetus; examined by culture in rat embryonic fibroblasts (REF). The virus produced typical herpesvirus-like CPE in REF inoculated with infected tissue homogenates beginning from 3 days p.i. and was identified as RCMV infection by IIP technique at day 5 p.i. The CPE and IIP results were similar as previously mentioned in Loh *et al *[19]. However, these features were not observed in mock-infected REF cells. The quantity of positive observations in different tissues is tabulated in Table 2.

**Table 2:** Positivity of CPE development and protein blotting of treatment groups in Experiment C and D.

**Test**	**Virus assay (CPE)**				**Protein blotting**			
**Experiment/Organ**	**C (Day 13–14 p.i.)**		**D (Day 21 p.i.)**		**C (Day 13–14 p.i.)**		**D (Day 21 p.i.)**	
	
	**Group**				**Group**			
	
	**v**	**pv**	**v**	**pv**	**v**	**pv**	**v**	**pv**

Uterus	5/5	5/5	5/5	5/5	5/5	5/5	5/5	5/5
Neonate	11/18	16/18	-	-	12/18	15/18	-	-
Placenta	-	-	16/20	10/10	-	-	14/20	8/10
Fetus	-	-	14/20	7/10	-	-	12/20	6/10
Embryo*	-	-	-	8/8	-	-	-	8/8

Note: Abbreviations: v = virus-infected and pv = virus-infected with immunosuppression.

* = eroded placenta and developing embryo in uterus at ≤ 7 days of pregnancy.

### Protein blotting

In the system, we used RCMV-infected cell lysate and mock-infected cell lysate, respectively for the positive and negative controls. The system was employed on the same samples for virus assay i.e. uterus and neonatal tissues collected from Experiment C as well as uterus, placenta and fetal tissues collected from Experiment D. The purified virus protein blots of uterus, placenta, embryo, neonate and fetus reacted positively in different frequency with HIS raised against RCMV (Table 2).

### PCR detection of *IE1 *gene

**Figure 3:**
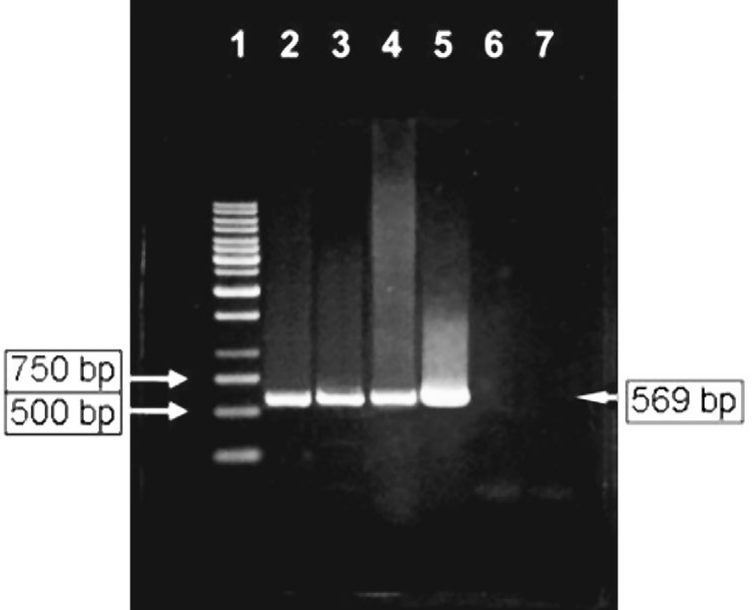
**PCR profile of *IE1*-specific products**. Viral DNA extracted from (i) infected immunosuppressed rats: uterus (C; day 14 p.i.; lane 2), 17-day old placenta (D; day 21 p.i.; lane 3), one-day post-partum neonatal tissues (C; day 14 p.i.; lane 4) and 17-day old fetal tissues (D; day 21 p.i.; lane 5); (ii) mock-infected immunosuppressed rats: uterus (C; day 13 p.i.; lane 6) and 17-day old placenta (D; day 21 p.i.; lane 7). Lane 1: GeneRuler™ 1 kb DNA ladder (Fermentas).

Similar samples tested in protein blotting were transversely analyzed by PCR amplification of viral DNA. Pure RCMV DNA serving as the positive control showed a distinct band of 569 bp in molecular size. Significant positive results in uterine, placental, neonatal and fetal samples were obtained (Figure 3). One heart sample, which had no immunostain in IIP test showed positive result in PCR. In contrast, no similar DNA band was detected in any tissue samples of control rats. The magnitude of positive observations is shown in Table 3.

**Table 3:** Positivity of PCR amplification of *IE1 *gene on viral DNA of treatment groups.

**Experiment/Organ**	**A (Day 21 p.i.)**		**B (Day 21 p.i.)**		**C (Day 13–14 p.i.)**		**D (Day 21 p.i.)**	
	
	**Group**							
	
	**v**	**pv**	**v**	**pv**	**v**	**pv**	**v**	**pv**
Brain	0/3	0/3	0/3	0/3	0/3	0/3	0/3	0/3
Heart	0/3	0/3	0/3	0/3	0/3	1/3	0/3	0/3
Testes	0/3	0/3	-	-	-	-	-	-
Ovary	-	-	0/3	0/3	0/3	0/3	0/3	0/3
Uterus	-	-	-	-	5/5	5/5	5/5	5/5
Neonate	-	-	-	-	12/18	15/18	-	-
Placenta	-	-	-	-	-	-	16/20	10/10
Fetus	-	-	-	-	-	-	14/20	8/10
Embryo*	-	-	-	-	-	-	-	8/8

Note: Abbreviations: v = virus-infected and pv = virus-infected with immunosuppression.

* = eroded placenta and developing embryo in uterus at ≤ 7 days of pregnancy.

0/3 = no detectable positive result over triplicate sample trials in all three rats.

### TEM examination

**Figure 4:**
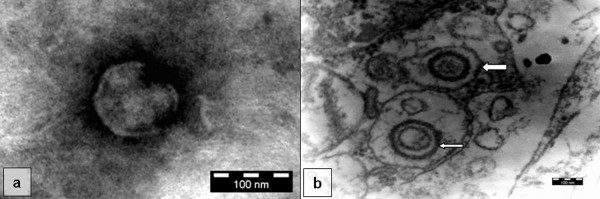
**Electron micrographs**. (a) negatively-stained herpesvirus-like naked nucleocapsid isolated from placenta sample of an infected immunosuppressed rat of 17-day pregnancy (D; day 21 p.i., × 168k), and (b) ultrathin sectioned placenta of the same rat (D; day 21 p.i.) showing enveloped virions with light capsid (thick arrow) and hollow core (thin arrow) present adjacently to nucleus and mitochondria (× 63k). All bar markers represent 100 nm.

TEM revealed virions exhibiting typical herpesvirus morphology in the placenta samples of the infected rats in Experiment D. None of the control groups established similar findings. Figure 4a shows the negatively stained naked virion with a size of about 106 nm. The virions were found either naked or enveloped (Figure 4b) in ultrathin section and mostly assembled near the mitochondria, golgi apparatus and endoplasm reticulum. The enveloped virions with a size of larger than 200 nm were found in a dense or light and sometime coreless capsid form.

### ELISA for antibody detection

**Figure 5:**
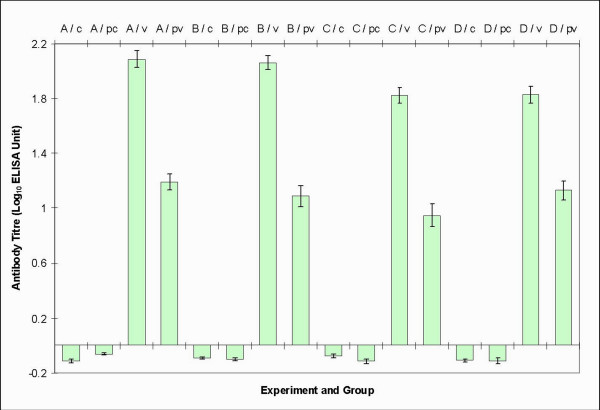
**The mean antibody titers of control and treatment groups in all experiments**. Abbreviations: c = mock-infected; v = virus-infected; pc = mock-infected with immunosuppression and pv = virus-infected with immunosuppression in Experiment A, B and D (day 21 p.i.); C (day 13–14 p.i.).

The humoral response of the animals at the end of the study is presented in Figure 5. The control groups of all experiments were devoid of RCMV-specific antibody. However, all the infected immunocompetent and immunosuppressed rats seroconverted and their antibody titers were significantly (*p *< 0.05) different to those of control groups. Meanwhile, the immunocompetent groups had significantly (*p *< 0.05) higher mean antibody titers than those of immunosuppressed groups.

### Fluorescent-antibody technique on buffy coat cells

The buffy coat cells of the two infected groups of rats in Experiment D were stained positively when observed under fluorescence microscope. Three categories of cells were differentiated based on their sizes, i.e. leukocytes, red blood cells and platelets in a descending order. The positive fluorescence-stained cells were the leukocytes of the infected rats especially those with immunosuppression.

## Discussion

The RCMV strain ALL-03 was first isolated from placenta and uterus of rats [19]. There was an urgent need to investigate and confirm the virus capability to infect the fetus. An attempt was made by Priscott and Tyrrell [12] to isolate RCMV from wild conceptuses. The failure of CPE observation during two weeks of culture concluded no evidence of transplacental infection in the single pregnancy of a naturally infected female [12]. However, in our study, an analogous procedure using conceptuses from Experiment D (about 7-day of gestation) was carried out. Interestingly, a delayed type CPE resembling characteristics that previously mentioned in our previous study [19] was observed.

Like HCMV, RCMV is poorly pathogenic in the immunocompetent host. The transient suppression in host immunity induced by cyclophosphamide is necessary for the induction of disease and the severity of disease always reflects the level of virus localization in the organs. The incubation time of symptomatic infection varied but commonly started at day 6 and onwards. This was similar to a previous study, which reported the emergence of clinical signs and absence of mortality in the immunocompromised groups [13]. The pregnant rats (Experiment C and D) seem to have partial immunosuppressive effect similar to that of other groups receiving cyclophosphamide as they were more permissive to RCMV infection than non-pregnant rats. Gould and Mims [20] showed that the virus could be reactivated during pregnancy. As a result of immunosuppression caused by the pregnancy alone or in conjunction with RCMV, the virus may have a better conducive environment for growth. In fact, one characteristic of CMVs is that the infection may have an immunosuppressive effect to the host during the acute phase. This has been observed in man, mice and rats [13,21,22].

Disease symptoms correlated well with the presences of infectious virus, viral antigen and DNA, which were found highest concentration in uterus, placenta, embryo and fetus; abundantly in lung, kidney, spleen and liver; less in salivary gland; even rare in heart (one case) but none in brain, ovary and testes. The detection of the RCMV in the spleen and liver was consistent with that of many previous studies [12,13,23,24]. The incidence of splenomegaly coincided with detection of RCMV in spleen. The finding is similar to that of mouse model [25]. The occurrence of RCMV immunoreactive monocytes and macrophages with characteristic inclusions in the spleen is consistent with the symptomatic infection. This parallels the situation in man where the involvement of the spleen is common in CMV infections [26]. The finding of RCMV particles in the liver parenchyma of immunocompromised rats is similar to that observed in HCMV infections, whereby the occurrence of hepatitis in immunocompromised patient is frequent [27]. The findings of the present study do closely resemble the pathological changes in the HCMV hepatitis, for example, the extensive liver damage with numerous inclusion bodies in hepatocytes, Kupffer cells as well as focal liver cell necrosis [28,29]. In our study, more viral antigens detected in the tubular epithelia than the glomeruli contrast to a previous study of RCMV strain Maastricht which localized predominantly in glomeruli and hardly ever in the tubular epithelia [24]. The finding that the renal capsule contained immunoreactive cells mimics that of the CMV infection in humans and rats [24].

Pneumonitis is the leading cause of death in CMV-infected transplant patients [14]. In RCMV-infected rats numerous immunoreactive cells were found in the lungs, including alveolar macrophages and interstitial mononuclear cells, resembling the histopathology of HCMV induced pneumonitis. Such damages caused by extensive virus replication in rats injected with cyclophosphamide are similar to that observed in the mouse model [30]. The virus persistence in the salivary glands resembles the typical characteristic of CMV in rat [23], mouse [25], guinea pig [31] and human [32]. The salivary gland is believed to be the principal route by which the virus is spread within the population of susceptible hosts [33]. The absence of a case in Experiment C may due to the fact that infectious RCMV (Maastricht strain) in salivary glands is detected at a later time than in all other organs, starting at day 14 p.i. [33]. In addition, the subcutaneous route and duration of infection (13–14 days) carried out in Experiment C would most probably decrease the severity of the disease. The submandibular gland was the preferred organ for tropism of the virus. These characteristics conformed to the previous study of Kloover *et al *[33].

The detection of viral antigen was not success in brain, heart, testes and ovary. Only one heart sample was found to contain viral DNA. This positive result was, most likely, due to contamination from infected blood cells. These four organs were reported to be involved in CMV infection in previous studies. A similar work studying acute infection of RCMV conducted previously [24] showed the brain tissue was negative for RCMV antigen. In contrast, a significant infection in brain was demonstrated in mouse model [34]. In fact, CNS involvement is a frequent feature of congenital infection [35]. MCMV infections were reported to be associated in the development of myopericarditis and dystrophic cardiac calcification [36] but cardiac infection in rat model was transient [13]. The recovery of infectious virus from sperm [37] and the detection of latent viral genomes in the prostate gland, testes, and spermatogonia of infected mice suggested that transmission of virus was by sexual contact [38,39]. With the congenital infection, inclusion-bearing cells are found also in testes and ovary after reactivation of latent infection. Nevertheless, the tropism of CMV in these germ line organs was in more chronic phase than the visceral organs [40]. Thus, it is reasonable to argue that the viral antigen as well as DNA of these germ line organs was untraceable.

The presence of RCMV infection in the endometrium of uterus regardless of pregnancy or different stages of pregnancy suggested that the uterus is one of the target organs. The current finding showed RCMV infection localized in different sites of uterus of different rats treated identically. One explanation might be that the different degree of susceptibility of an individual to the infection by which is largely affected by the host's physiology and immune response. Besides, CMV is evident by its asynchronous development *in vitro *[41]; it might also happen *in vivo*. The uterine infection extends to adjacent cell type during more advanced dissemination, i.e. from stromal cells to epithelial cells. This observation is similar to CMV infection in human and contiguous endometrial cells dissemination plays an important role in congenital infection where HCMV can establish active and latent infection to the placenta subsequently [3].

High un-natural dosage of infection at titer 10^6 ^TCID_50 _per rat has no effect on abortion and severe fetus wastage as observed in Experiment C and D. These findings contrast to guinea pig CMV infection by which the highest rates of fetus resorption/abortion and mortality are correlated well with the increase of infection dosage [42]. This might suggest that RCMV strain ALL-03 is either a benign virus for the offsprings naturally or somewhat attenuated throughout the subsequent tissue culture passages or when infecting a different rat strain. If the attenuation of tissue culture passage is the case, it can be reversed by a few *in vivo *passages and the pathogenicity of this 'virulent' virus can be determined in future investigation. On the other hand, one explanation, which is more fascinating, might be that the current experiments performed using a virus isolated from the black rat, *Rattus rattus diardii*, in a laboratory rat, *R. norvegicus*. This different host strain may contribute to the mild effects of the fetal and neonatal infections. Nevertheless, a definite answer for this speculation cannot be given presently since we realize that there is no SPF colony of *R. rattus *available for the moment.

Although virus infection in Experiment C was conducted via s.c. route (less infective than i.p. route) and in shorter incubation period (about 13–14 days), the signs of infection were closely resembling those of Experiment D. These indicate maternal virus dissemination had started earlier than 2 weeks time. The *in utero *virus transmission was more promising when one-day old neonates and conceptuses (fetuses and embryos) had already harbored the virus. In fact, there was no probable virus transmission from the female rats to them perinatally or postnatally by close contact. This is due to the slow growth of RCMV which is normally detected in organs such as kidney and salivary gland starting on day 4 and 10 p.i., respectively [12,13]. Therefore, it is believed that the virus transmitted either by direct passage of the virus across the placenta to the fetus or through germ cells as proposed by Brautigam and Oldsone [43], Chantler *et al *[44], and Osborn [45]. However, the precise localization of the virus in tissue section for IIP test had elucidated that the infections occurred in placenta, uterus, embryo, fetus and neonate, but not in testes and ovary. The presence of infectious viruses in the aforementioned sites suggests the RCMV infection was successive and responsible for the vertical transmission. Furthermore, electron microscopy showing visible typical herpesvirus-like particles in infected placenta, had further confirmed the transplacental transmission route of RCMV strain ALL-03 without doubt. Generally, the frequency and concentration of virus infection were predominantly in the uterus, placenta and offspring differing from those reported previously in other RCMVs. It is believed that this unique infection preference was indeed the nature of ALL-03 virus.

The presence of CMV infection in the placental parenchyma and membrane had been confirmed in a previous study [5]. It is likely that CMV or CMV DNA could be detected in the villi, including the mesenchyme and trophoblasts, extravillous trophoblast, and decidual cells. Consistent with their study [5], the IIP staining in our study showed immunogenic sites containing RCMV antigen were the decidual basalis, junctional and labyrinth zones. The likely cells involved were the trophoblast and decidual cells. The placenta of earlier gestation (about 7 days) showed more signs and intensities of infection than that with lengthier gestation period. Furthermore, the intensity of infection in placenta surpassed that in fetal tissues. However, at later stage (> 14 days) of pregnancy, the fetal tissues such as liver and kidney showed a more significant infection. The most likely explanation of the events might be the differences in the degree of permissiveness to RCMV in various tissues during development. The virus may subsequently infect the fetus following direct crossing of the labyrinth zone of placenta after a successive virus replication period.

The exact mechanism of how RCMV crosses the placenta to infect fetus has yet to be elucidated. It is either caused by viremia, transportation of the virus by maternal leukocytes entering the placenta, direct passage of the virus from uterus into the placenta or direct invasion of placenta and fetus. The preliminary study employing immunofluorescence staining on buffy coat cells of rats in Experiment D found that the infected rats suffered a leukocyte associated-viremia. The circulation of infected leukocytes in the blood had most probably promoted the spread of the virus throughout the animal body. This finding was in agreement with that of Bruggeman *et al *[13]. Similar observation had also been made for HCMV [46]. During the viremic phase, the virus circulates and disseminates as it has been carried in leukocytes [47].

The findings obtained from Experiment D with absolute uterine infection in relation to 70–100% of placental infection illuminate the important intersection of maternal uterus for the congenital infection. Indeed, the earlier *in vitro *study in which leukocytes infected with a clinical HCMV strain VR1814 (thus reproducing the *in vivo *phase of acute viremia) was used to infect either explants of floating and anchoring villi or differentiating cytotrophoblast cells, no infection was observed [6]. On the other hand, the same study showed that HCMV-infected leukocytes could productively infect uterine endothelial cells, which in turn, were able to transmit the infection to cytotrophoblast cells. In this context, the infected anchoring villi, which extended into the uterine wall passing the infection to the placenta, were well demonstrated in our study. Concurrent to the aforementioned *in vitro *model and our findings, congenital infection is acquired only during primary maternal infection whereby uterine infection must take place preconceptionally or periconceptionally.

Another intriguing aspect of the natural history of HCMV infection during pregnancy concerns the transmission rate during different gestation period. In particular, while primary HCMV infection acquired either before or around conception carries the lowest risk of transmission [48], maternal infections acquired during the first and second trimester of gestation can be transmitted at a similar rate (approximately 45%). On the other hand, during the third trimester, maternal infection has the highest probability of being transmitted to the fetus (78.6%). These data clearly indicate that: (i) the virus is transmitted efficiently from mother to fetus despite the presence of an innate barrier; (ii) mechanisms of protection are more effective during the first two-thirds of gestation, becoming less effective in late pregnancy [49]. In parallel to these reports, the dams of Experiment D and Experiment C by which infections occurred in preconception and during the midterm (about 10 days) showed transmission to offsprings in 65–81% and 59–84%, respectively. It agreed that the infection to offsprings was more effective in dams of Experiment C occurring in earlier time interval (day 13–14 p.i.). Since placental infection has been detected either in the presence or absence of fetal infection, the placenta is considered as the most important site of either protection (by sheltering the fetus from CMV infection) or transmission (by acting as a viral reservoir and allowing the infection to reach the fetal compartment). Nevertheless, whenever an infection of the fetus occurred, virus could be found in the associated placenta at different degree of infection. Moreover, the discrepancy of the number of positive virus infection in placenta to that of fetus was only 10–30%. Hence, it is suggested that the placenta more likely serves as a reservoir rather than protective barrier in which the virus replicates first prior reaching the embryo or fetus in our study. As discussed earlier, the human placenta is not an effective barrier to HCMV transmission in the same way [3].

## Conclusions

The current study exhibits a widespread systemic RCMV infection. The maternal viremia, uterine infection, placental infection and direct dissemination to the fetus are the most likely sequence of events leading to congenital infection after a primary maternal infection mimicking the features of congenital CMV infection in human. We believe that RCMV strain ALL-03 has the potentials to provide predictable information on the pathogenesis and manifestations of congenital CMV infection, rational designs of new antiviral therapies as well as *in utero *vaccine to specifically prevent prenatal infection in future investigations.

## Methods

### Preparation of virus working stock and hyperimmune serum (HIS)

Virus stock of RCMV strain ALL-03 was prepared and titrated by mean of TCID_50_/ml prior to animal inoculation. Hyperimmune serum (HIS) was prepared in mice according to standard procedure using heat-inactivated purified RCMV suspension (10^7 ^TCID_50_/ml). The antibody titers were determined using indirect enzyme-linked immunosorbent assay (ELISA).

### Design of the experiments

Two-month old SPF Sprague-Dawley rats were assigned into four different experiments (A, B, C and D). The rats from each experiment were subdivided into immunocompetent and immunosuppressed groups. Each immunosuppressed rat was induced by subcutaneous (s.c.) injection with cyclophosphamide at a dosage of 40 mg, a day before the virus inoculation. All treatment groups were infected with 10^6 ^TCID_50 _RCMV suspensions in either intraperitoneal (i.p.) or s.c. route. Five rats were allotted for each treatment group, whereas, three rats which inoculated with PBS in similar route served as the control group. Blood samples were collected before and after the experiments for antibody titration. The animals were observed twice daily for clinical signs and mortality.

The male and non-pregnant female rats employed in Experiment A and B respectively, were inoculated i.p. route with RCMV suspensions. These rats were sampled at day 21 p.i. The brain, salivary gland, heart, lung, spleen, liver, kidney, testes and uterus were processed for hematoxylin and eosin (H&E) staining, and indirect immunoperoxidase (IIP) test.

In Experiment C, female rats of about 10-day pregnancy (determined by vaginal plug observation) were inoculated via s.c. route. The inoculation was carried out in s.c. route rather than i.p. in order to prevent abortion that may be caused by the injection. The sampling was carried out at one day post-parturition of the neonates, i.e. day 13–14 p.i. of the dams. The brain, salivary gland, heart, lung, spleen, liver, kidney, uterus, ovary and the one-day old neonatal tissues (salivary gland, liver and kidney) were subjected to H&E staining and IIP test. Additional neonatal tissues and uterus were assigned for virus assay to isolate the infectious virus as determined by cytopathic effect (CPE) development (as described in Loh *et al *[19]); protein blotting as well as polymerase chain reaction (PCR) amplification.

In Experiment D, non-pregnant female rats were inoculated via i.p. route. The rats of each group were housed together with a male rat and observed for pregnancy. The pregnant rats were sacrificed at day 21 p.i., i.e. just before delivery. The salivary gland, heart, lung, spleen, liver, kidney, uterus, ovary, placenta and fetal tissues (liver and kidney) were prepared for H&E staining and IIP test. Additional uterus, placenta and fetal tissues were further tested by virus assay, protein blotting and PCR analyses. The remaining placenta was processed for transmission electron microscopy (TEM) examination.

### Indirect immunoperoxidase (IIP) test

After deparaffinization, the sections were blocked for endogenous peroxidase and covered with 1% SDS/PBS for 5 minutes. Following washing thrice with PBS containing 1% Triton X-100 (PBSTx), the sections were immersed in 5% BSA/PBSTx for 1 hour. The diluted mouse HIS (1:200) with additional 2% normal rat serum (only for neonatal tissues) was added and incubated for 1 hour at 37°C. The sections were washed and incubated with diluted peroxidase-conjugated goat anti-mouse IgG (1:250). After stopping the stain development of DAB substrate (KPL), the sections were counterstained with hematoxylin, washed in dH_2_O, dehydrated and then mounted.

### Protein blotting

The test strips (Millipore) pre-treated with transfer buffer, were blotted with purified intracellular virus (from tissue homogenates). The air-dried test strips were immersed in 5% BSA/PBS and then incubated with diluted mouse HIS (1:500) for 1 hour. After washing in PBST (0.2% Tween 20), the test trips were incubated with diluted peroxidase-conjugated goat anti-mouse IgG (1:2000) for 1 hour and then washed again. DAB substrate was added. The test strips were rinsed in dH_2_O and then air-dried.

### Polymerase chain reaction (PCR)

The sequences of the gene-specific primers flanking on immediate-early 1 (*IE1*) gene region of RCMV strain ALL-03 were 5'-CACAGAGATCTCACTAACCTGCCACCTATAACCAC-3' (Forward) and 5'-TCCAGCAGACTTCTGTATCCTGATTCAAG-3' (Reverse). The PCR reaction contained 100 ng DNA extracted from each tissue sample, 0.5 μM of each primer, 1X optimized buffer, 0.2 mM dNTP mix, 2 unit of DyNAzyme™ II DNA polymerase (Finnzymes) and nuclease-free H_2_O. The protocol included an initial denaturation step at 95°C for 5 minutes, 40 cycles of 1-minute denaturation at 94°C, 30-second annealing at 69°C and 1-minute extension at 72°C. This was followed by a final extension step at 72°C for 1 minute.

### Transmission electron microscopy (TEM) examination

Intracellular virus from placenta was purified and subjected to negative staining. For ultrathin sectioning, the placenta was processed accordingly to the procedures described in Loh *et al *[19] and subjected to TEM examination.

### Indirect enzyme-linked immunosorbent assay (ELISA)

Pre-immune and hyperimmune sera were used as negative and positive controls, respectively. Microtiter plates (Dynatech) were coated with purified virus (3.2 μg/ml). Reaction wells were rinsed thrice with PBST (0.05% Tween 20 in PBS) and blocked with 5% BSA/PBST. After incubation with diluted test sera (1:50) at 37°C for 2 hours, the bound antibodies were reacted with diluted peroxidase-conjugated goat anti-rat IgG (1:2000) for another 2 hours. Following washings, TMB substrate (KPL) was added. The absorbance of a sample was determined using an ELISA reader.

### Statistical analysis

Data were expressed as mean ± SD, and statistical analysis was performed using two-tailed Student's *t*-test. Differences between groups were considered statistically significant at *P *< 0.05.

### Fluorescent-antibody technique on buffy coat cells

A test to assess cell-associated viremia was conducted on buffy coat cells of animals in Experiment D. The buffy coat cells were fixed on a chamber slide and subjected to an indirect immunofluorescence (IIF) procedure as mentioned in Loh *et al *[19] with a few modifications, i.e. using mouse HIS at dilution 1:200 (in 1% BSA/PBS) and FITC-conjugated goat anti-mouse IgG at dilution 1:250. The normal mouse sera were used as negative controls.

## Abbreviations

cytomegalovirus (CMV), cytopathic effect (CPE), enzyme-linked immunosorbent assay (ELISA), hematoxylin and eosin (H&E), hyperimmune serum (HIS), immediate-early 1 (*IE1*), indirect immunofluorescence (IIF), indirect immunoperoxidase (IIP), intraperitoneal (i.p.), murine cytomegalovirus (MCMV), polymerase chain reaction (PCR), post-infection (p.i.), rat cytomegalovirus (RCMV), rat embryonic fibroblast (REF), subcutaneous (s.c.), transmission electron microscopy (TEM).

## Competing interests

The author(s) declare that they have no competing interests.

## Authors' contributions

HSL participated in the experimental design, performed all experiments and drafted the manuscript. MAML participated in the experimental design and coordination and helped to draft the manuscript. SOAR conceived of the study and participated in its design and interpretation of data. LJK participated in part of the experiments and assisted in post-mortem investigation. All authors read and approved the final manuscript.
